# Camera trocar lifting in office gasless laparoscopic sterilization under local anesthesia

**DOI:** 10.3109/00016349.2010.486435

**Published:** 2010-05-05

**Authors:** Bo S. Bergström

**Affiliations:** Department of Obstetrics & Gynecology, Nordfjord Hospital, Nordfjordeid, Norway

**Keywords:** Female sterilization, laparoscopy, office, gasless, local anesthesia, surgical techniques, endoscopic surgery

## Abstract

We evaluated 35 cases of a mechanical approach to abdominal wall lifting, used in office-based gasless laparoscopic sterilization under local anesthesia. Lifting of the abdominal wall, using the camera trocar as an anchoring device and complemented by suprapubic lifting by means of a towel clamp, led to passive intra-abdominal air filling, giving sufficient space to identify, anesthetize, coagulate and cut the Fallopian tubes. Only mild sedation was necessary. All women walked to and from the operating room. All had successful tubal ligation. The overall satisfaction rate was 97%. The mechanical lifting moment was not painful. With the exception of one woman with failed tubal anesthesia, all women had a low mean pain score of 2.6 (VAS 0–10). No complications occurred except one wound infection. The costs were < ¼ of those of traditional laparoscopic sterilization and office hysteroscopic sterilization. This approach is effective for office-based laparoscopic sterilization. Room air, two strings and a needle replace active gas insufflation and narcosis.

## Introduction

Modern healthcare is expensive, especially surgery performed in a regular operating theatre. General anesthesia involves life-threatening risks and undesirable side-effects. Local anesthesia minimizes these drawbacks and can be administered by the surgeon. This promotes simplification.

Female sterilization in an office situation using laparoscopy with a low-pressure pneumoperitoneum administered under local anesthesia has been performed for more than 30 years (1), but has not gained widespread popularity. A survey of the American Association of Gynecologic Laparoscopists (2) showed that only 5.1% of its members performed office-based laparoscopy under local anesthesia. This is a low incidence for doctors with a special interest in laparoscopy. One explanation can be that active insufflation of gas gives too much pain and necessitates heavy conscious sedation and surveillance by specially trained personnel (3). Even a low-pressure pneumoperitoneum is painful. By contrast, a zero-pressure (gasless) pneumoperitoneum is painless. Office-based laparoscopy and gasless laparoscopy are not new procedures, but gasless laparoscopy under local anesthesia is a new procedure. Two articles have been published on gasless laparoscopic female sterilization but these operations were done under general anesthesia (4,5).

Carbon dioxide gas is cold, very dry, dissolves in water and provokes hypothermia, desiccation, tissue irritation, and acid-base and blood gas changes. By comparison, room air is warmer, humid, insoluble, not irritating and available everywhere. Gasless laparoscopy depends on mechanical lifting of the abdominal wall with a passive inflow of room air. An open access technique for trocar insertion is safer than a closed access technique and is therefore a better choice for office laparoscopy (1). The open access technique results in a 1.5–2 cm wide hole in the abdominal wall, so using a micro-laparoscope is meaningless. An ordinary laparoscope has advantages in focal distance and field of vision.

## Material and methods

This was a prospective pilot study to evaluate a mechanical approach to lifting, used for office-based gasless laparoscopic sterilization. Women with a body mass index (BMI) < 30 kg/m^2^, no serious illnesses and no known abdominal adhesions were informed about general and local anesthesia during presterilization counseling sessions. All women without contraindications for office surgery chose local anesthesia and were given written information about the procedure. Between September 2003 and December 2005, 35 women were sterilized in a low resource setting situated one floor below a regular operating theatre. These women had a mean age of 39 years (range 27–48), BMI 24.1 kg/m^2^ (19–30). Fourteen women had had a previous abdominal operation. All women but one had a normal-sized uterus. The procedure room had resuscitation equipment including oxygen and suction, an emergency tray with diazepam (5 mg/ml), atropine (1 mg/ml), catastrophic adrenaline (0.1 mg/ml) and a narcotic antidote, electrocardiography equipment, pulse oximeter and an alarm button.

Women had fasted for at least 6 hours and were premedicated orally with 200 mg ibuprofen and 1 g paracetamol/30 mg codeine phosphate about 1 hour before the start of surgery. After voiding, each patient walked to the procedure room where she was given intravenous fluid. Personnel included the surgeon, an assistant to arrange the instruments and a midwife to monitor the patient's blood pressure, pulse rate, respiratory rate, blood oxygen saturation, electrocardiogram, level of sedation and to give medications on request from the surgeon. Only the surgeon was dressed in sterile clothing. The patient was cleaned by the assistant and draped by the surgeon and mildly sedated with 5 mg diazepam and 25 mg meperidine: doses that could be repeated once. The muscle relaxant effect of diazepam was of value in the mechanical stretching of the abdominal wall. A mixture of 40 ml 1% lidocaine hydrochloride/adrenaline and 60–80 ml 0.9% sterile NaCl was used for local anesthesia. The anterior region of the cervical portio was anesthetized and a Hulka forceps attached. The abdominal wall just beneath the umbilicus was anesthetized and a >12.5-mm trocar was inserted into the abdominal wall using an open access technique. The lifting technique used the camera trocar as an anchoring device in the abdominal wall. The open trocar gas inlet allowed a free inflow of room air. An operative (0°, 10 mm) laparoscope with a 6-mm working channel was used. The trocar/abdominal wall were lifted with a loop of polydioxanone suture (PDS #1) snared around the shaft of the trocar with a hang knot and needle-driven through the fascia, cutaneous tissue and skin in the lower end of the abdominal wall incision. The loop suture was attached to a horizontal metal arm mounted on the operating table and placed above the woman.

Padded shoulder supports were an important prerequisite, because if a woman were to slide downwards on the table she would be likely to get scared and tense her abdominal muscles to stay put. This would tend to press the intestines into the pelvis. However, despite the shoulder supports it was necessary for the women to lie with their pelvic region 10 cm outside the table. This position gave necessary sliding distance to prevent the Hulka forceps from being blocked by the table when it was placed in steep Trendelenburg position.

Mechanical lifting of the abdominal wall with the camera trocar as an anchoring device and with the trocar gas inlet open, led to passive filling of the abdomen with air. Lifting the skin and subcutaneous tissue 6–8 cm above the symphysis pubis in the same way using a towel clamp allowed sufficient air into the abdomen for laparoscopic sterilization. The combination of mechanical lifting, the Trendelenburg position and the forward rotation of the uterus created adequate intra-peritoneal space to identify, anesthetize, coagulate and cut the tubes. If the mechanical lifting procedure did not create sufficient space to identify the tubes, a small amount of additional room air (1) was insufflated actively using a rubber bulb. A towel clamp closing the upper end of the abdominal wall incision then secured air tightness. The mechanical lifting supported most of the weight of the abdominal wall and only a small volume of actively insufflated air and a low intra-abdominal pressure increase were needed to create additional space. After the tubes had been anesthetized, coagulated, divided with hook-scissors and the intra-peritoneal air been reduced to a minimum, the abdominal wall opening was closed. The women were able to sit up for a minute and then walk back to the recovery room.

All women received postoperative information from the surgeon or the midwife. They were given oral medication for 2 days (200 mg ibuprofen × 3 and 1 g paracetamol/30 mg codeine phosphate × 3) and a questionnaire to be sent back in 1 week (pain score, worst painful moment, satisfaction rate, complications, validity of the presterilization counseling session).

## Results

All women had a successful tubal ligation. The overall satisfaction rate was 97%. Two of the first 10 women in the study had a small amount of filtered room air insufflated actively, but when the camera trocar lifting procedure was complemented by supra-pubic lifting, there was no need for active air filling because the intra-abdominal laparoscopic view was always good. No woman reported the mechanical lifting moment to be painful: one stating, ‘It was like being lifted in the pants'. Thirty-four of 35 women were satisfied in that they answered ‘yes’ to the question of whether they would recommend the same operation to their best friend. One woman was not satisfied; the reason was much pain when one of the tubes was electro-coagulated. This was the only woman to report that the interprocedural pain was worse than was expected from the information given in the presterilization counseling. Per/postoperative pain was measured using a visual 0–10 analog scale (VAS). The 34 satisfied women reported an average score of 2.6 (range 0–7.5) and three expressed no pain at all. The potentially painful moments during the operation were the different needle pinpricks, the Hulka forceps manipulations, unintentional rough touching of pelvic organs and anesthetic failure. One woman reported VAS 7.5 for the steep Trendelenburg position and one woman VAS 7.0 for the Hulka forceps application. Minor sedation was sufficient. All women walked to and from the operating room. No operation was converted to general anesthesia and there were no complications except for one wound infection. All women left for home after 1–5 hours and all submitted a completed outcome questionnaire.

## Discussion

In the first 10 operations, a 1.2-mm puncture needle was used for tubal anesthesia. This needle had a tendency to push the tube in front of itself rather than to penetrate. Later, a 0.4-mm needle was used. When using a 10-mm laparoscope, a >12.5 mm camera trocar with an open high-flow gas inlet is necessary for the free passive inflow of room air. A smaller trocar can reduce the inflow of air, and result in a negative intra-abdominal pressure, that limits the space for inspection and instrumentation and causes pain. The used mechanical lifting procedure has a short setup time (< 1–2 min), introduces no extra devices into the peritoneal cavity, causes no trauma to the peritoneal surface and does not interfere with surgical movements.

**Figure 1:**
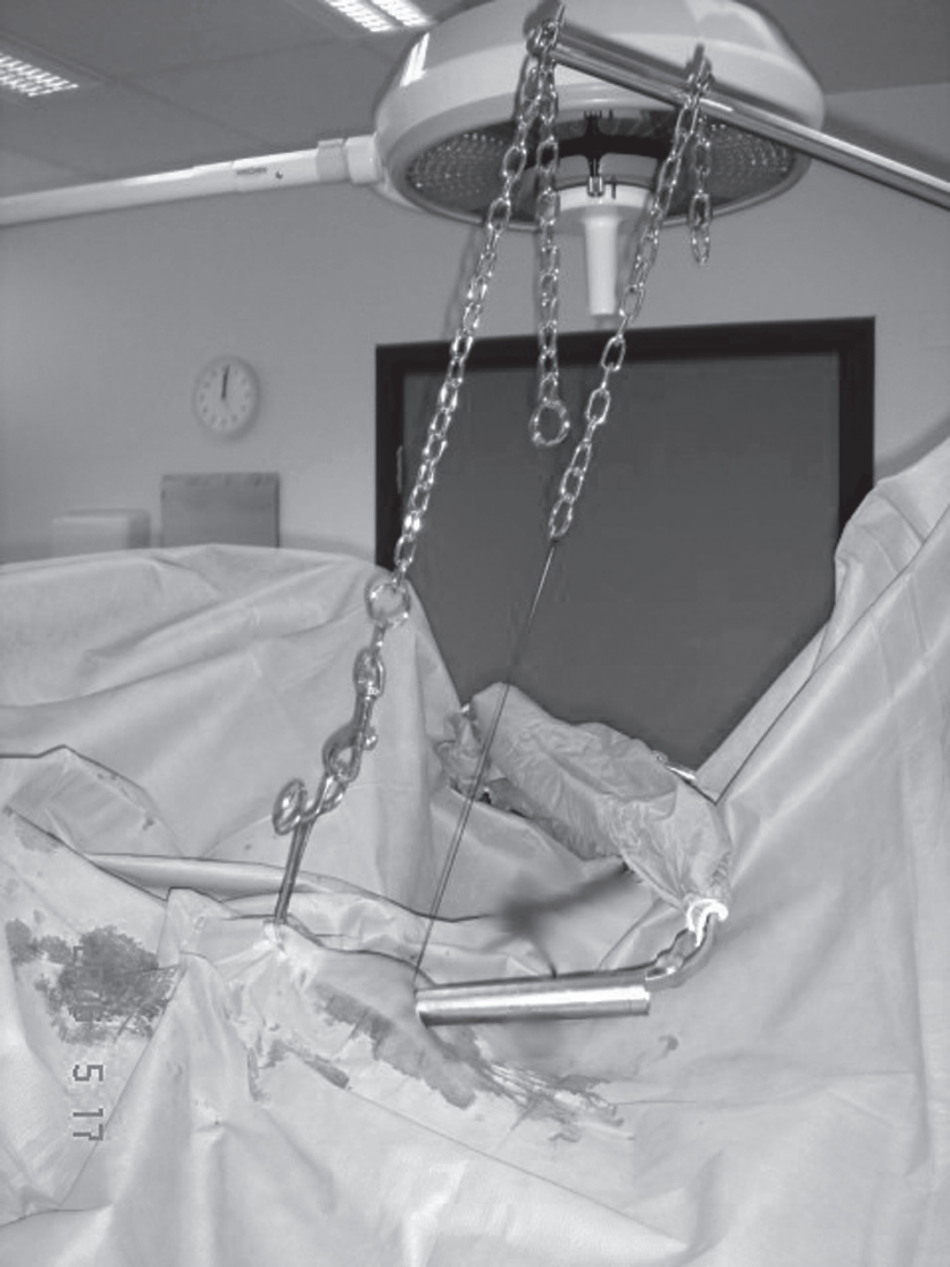
Mechanical lifting

To further simplify the process, the surgeon can use an amnioscope (20 × 200 × 25 mm), which permits direct visual inspection and instrumentation of the tubal areas (Figure 1). Short instruments are then used for an optimal visual distance. Since 1993, the author has performed more than 200 female sterilizations using an amnioscope. The only drawback compared to using a laparoscope are a less comfortable working position for the surgeon and the fact that the personnel and patient cannot watch the procedure on a monitor. An amnioscope with or without an operative laparoscope is a good choice, that supports a high inflow of room air and to insert a second working instrument beside the laparoscope to displace obscuring loops of distended bowel, if any.

Using an amnioscope and operate under local anesthesia and mild sedation in an office setting conforms well to the statement of the WHO Task Force on Female Sterilization (6). ‘The ideal female sterilization would involve a simple, easily learned, onetime procedure that could be accomplished under local anesthesia and involve a tubal occlusion technique that caused minimum damage. The procedure would be safe, have high efficacy, be readily accessible, and be personally and culturally acceptable. The cost for each procedure would be low and there would be minimal costs for the maintenance of equipment'.

In developing countries, where minilaparotomy is a common approach, women would probably benefit from this amnioscope lifting procedure. Compared with minilaparotomy, it gives a much better view of the pelvic organs, an aesthetically more acceptable scar, a shorter recovery period and less complications/ complaints (6). Using an ordinary anesthesia frame in the lifting process, with the horizontal arm draped in a sterile sleeve, makes the procedure progress even more smoothly. In developing countries, where resources are limited for the purchase and maintenance of more sophisticated laparoscopic equipment, this amnioscope lifting procedure is truly a cheaper and safer option for female sterilization than the traditional laparoscopic procedure presently used in the developed world.

Office laparoscopic sterilization requires, in contrast to office hysteroscopic sterilization, no scheduling of surgery according to the woman's menstrual cycle and has an almost 100% first-attempt success rate, an immediate effect on fertility, no need for tubal patency control, no material costs, a reversal success rate of 55–75% (7), an unchanged possibility of IVF and can be performed within 48 hours of delivery.

Office laparoscopy is claimed to cost much less than traditional laparoscopy. In one study, there was an almost 80% reduction in costs (8), which is in agreement with the present study. Our total costs in 2006 were calculated to be NOK 2,895 (US $446), which represents a 75% reduction. Gasless laparoscopic sterilization and hysteroscopic sterilization (Essure® device) can both be done in an office-based setting with the same OR-team and operation time. The Essure procedure is however approximately US $1,575 more expensive due to additional costs for the Essure® devices (ESS305, Conceptus, Inc., USA, Retail Price: $1,299) and for tubal occlusion control (HSG US $275) and is contraindicated within 6 weeks after delivery and in women with hypersensitivity to nickel or allergy to contrast media.

A comparative study/literature review of hysteroscopic sterilization versus laparoscopic tubal sterilization (9) showed overall standard complication rates for laparoscopic sterilization of 0.8–0.9% (6,9) and a major complication rate for hysteroscopic sterilization of 3.2% (9). Moreover, correct use of local anesthesia removes the single greatest source of risk in conventional laparoscopic sterilization procedures, general anesthesia.

**Figure 2:**
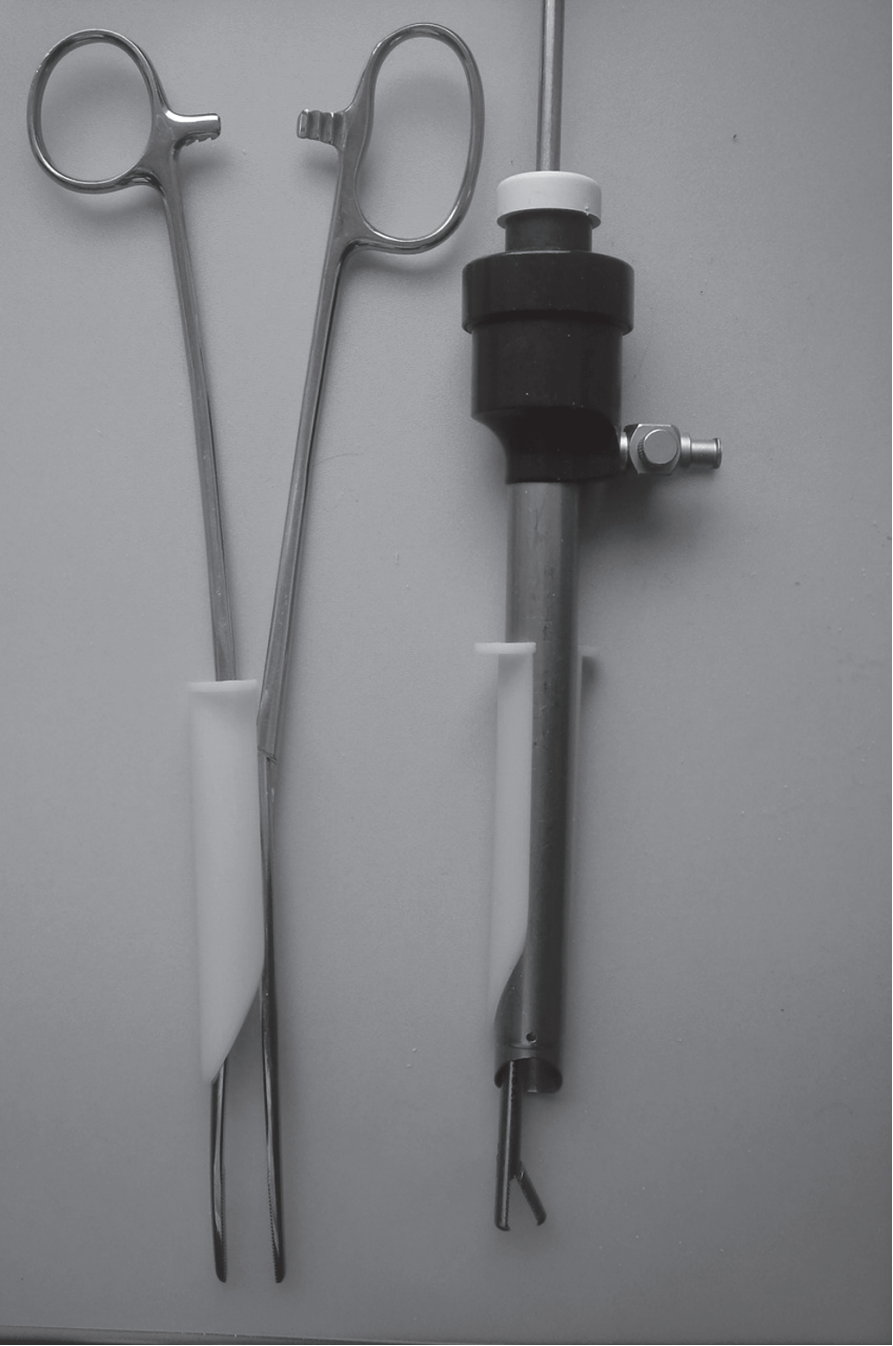
Slit-trocar for gas/gasless laparoscopy

A gastight mechanical lifting procedure, that creates adequate intra-peritoneal space for female sterilization under local anesthesia, is also useful in surgery under general anesthesia. Therefore, in conventional gas laparoscopy, the author also uses the described lifting technique. Such lift-assisted laparoscopy makes surgery in low gas pressure (1–6 mm Hg) possible with sustained optimal or adequate view (10) and, in case of need, to take temporary measures in a ‘gasless’ condition. An immediate shift between low, standard and zero gas pressure is possible. With no gas pressure, conventional open surgery instruments such as clamps, scissors and powerful suction devices can be used and with standard gas pressure, the complementary abdominal wall lifting means a ‘double’ outcome in forming the intra-abdominal space. A special slit-trocar facilitates the shifting maneuver between the gas-based and gasless technique (Figure 2). The finding that the centrally positioned camera trocar, except for its normal function as a gastight sleeve for the laparoscope, is also a perfect anchoring device for mechanical lifting, is an enhancement for laparoscopic surgery. ‘Yesterdays’ gasless laparoscopy must not be confused with lift-assisted laparoscopy. The European Association for Endoscopic Surgery states that ‘gasless laparoscopy has no clinically relevant advantages compared to low-pressure (5–7 mm Hg) pneumoperitoneum’ (11). There may be two exceptions to this: laparoscopy under local anesthesia and lift-assisted laparoscopy under general anesthesia if it is required to use a conventional open surgery instrument. All patients benefit from low-pressure gas laparoscopy and especially high-risk patients, including pregnant women.

The evaluated mechanical lifting technique can be applied effectively in office laparoscopic sterilization, even to overweight women. Risks of general anesthesia and active gas insufflation are eliminated and room air, two strings and a needle, replace a CO_2_ gas filling machine and narcosis
